# A Novel PCCA Mutation in a Patient With Late-Onset Propionic Acidemia Identified by Genetic Diagnosis Panel

**DOI:** 10.3389/fped.2018.00233

**Published:** 2018-08-21

**Authors:** Yanyun Wang, Yun Sun, Tao Jiang

**Affiliations:** Center of Genetic Medicine, The Affiliated Obstetrics and Gynecology Hospital of Nanjing Medical University, Nanjing Maternity and Child Health Care Hospital, Nanjing, China

**Keywords:** Propionic academia (PA), PCCA gene, tandem mass spectrometry (MS/MS), urine gas chromatography mass spectrometry (GC/MS), Genetic diagnosis panel

## Abstract

**Background:** Propionic acidemia (PA) is an extremely rare autosomal recessive disorder which is caused by the deficiency of propionyl-CoA carboxylase (PCC) and associated with pathogenic variants in PCCA or PCCB gene.

**Case Report:** Detection of PA in neonates is possible using Propionyl carnitine (C3) analysis by tandem mass spectrometry (MS/MS) in dried blood spots (DBS). Here we report one patient with PA. C3 in this case was normal in the initial screening and recall check and only manifested as the slightly increase of C3/C2, 3-hydroxypropionate in urine was only slightly elevated. Then two pathogenic mutations (c.802C>T/c.827delG) were detected in the PCCA gene by Genetic diagnosis panel. Among them, the variation rs774738181 (c.802C>T) was present on the dbSNP database which appeared to be “Likely pathogenic” in GenBank dbSNP (100915068). c.827delG was a novel frameshift mutation, leading to p.Gly276ValfsX46 mutation of amino acid sequence in PCCA. The patient underwent 1 year of follow-up, had total of 7 times and remain asymptomatic whose blood ammonia and liver function were normal. When the child was 1 year of age (in May of 2017), C3 and 3-Hydroxypropionate sudden elevated significantly, that proved pathogenicity of c.802C>T and c.827delG.

**Conclusion:** Two novel mutations (c. 802C>T and c.827delG) in PCCA gene may be associated with late-onset PA, expanding its mutational spectrum. Maybe there is relation between the severity of propionyl-CoA carboxylase (PCC) activity defects and different genotypes.

## Introduction

Since the first report of Propionic Acidemia (PA) by Hommes in 1968 (1), PA has been investigated by the researchers around the world. PA is an autosomal recessive genetic organic acidemia due to propionyl-CoA carboxylase (PCC) activity defects, leading to the abnormal accumulation of propionic acid and its metabolite precursor.

PCC is α6β6 multimer composed of α and β subunits, the coding genes of α and β two subunits are PCCA and PCCB, respectively (2). In this study, we reported one patient with PA who was screened by tandem mass spectrometry (MS/MS) and diagnosed by urine gas chromatography mass spectrometry (GC/MS), then we used Genetic diagnosis panel to analyze the patient and identified a novel mutation in PCCA gene.

## Case report

The child, a Chinese boy with birth weight of 3,450 g and gestational age of 39^+6^ week (his mother was 28-year-old, G1P1, with normal pregnancy), was screened in Genetics and Metabolism Department of the Obstetrics and Gynecology Hospital affiliated to the Nanjing Medical University at 3th day after birth. The results are shown in Table 1. MS/MS showed that he had elevated C3/C2 but C3 and 3-hydroxypropionate remained almost normal. In order to identify the etiology, the patient and his families were diagnosed by Genetic diagnosis panel in our hospital on 16 June 2016. Parents were healthy and non-consanguineous.

**Table 1:** The results of MS/MS and GC/MS in neonatal period.

**DATE**	**MS/MS**				**GC/MS**
	**C0 (10–50 μmol/L)**	**C3 (0.38–3.6 μmol/L)**	**C3/C2 (0.04–0.2)**	**C3/C0 (0.02–0.17)**	**3-Hydroxypropionate (0–1.1)**
2016.05.23	24.59	3.22	0.39 ↑	0.13	—
2016.05.31	17.62	3.41	0.42 ↑	0.19 ↑	—
2016.06.06	19.78	3.33	0.44 ↑	0.17	3.21
2016.06.16	26.21	3.96 ↑	0.43 ↑	0.15	3.11

**Figure 1:**
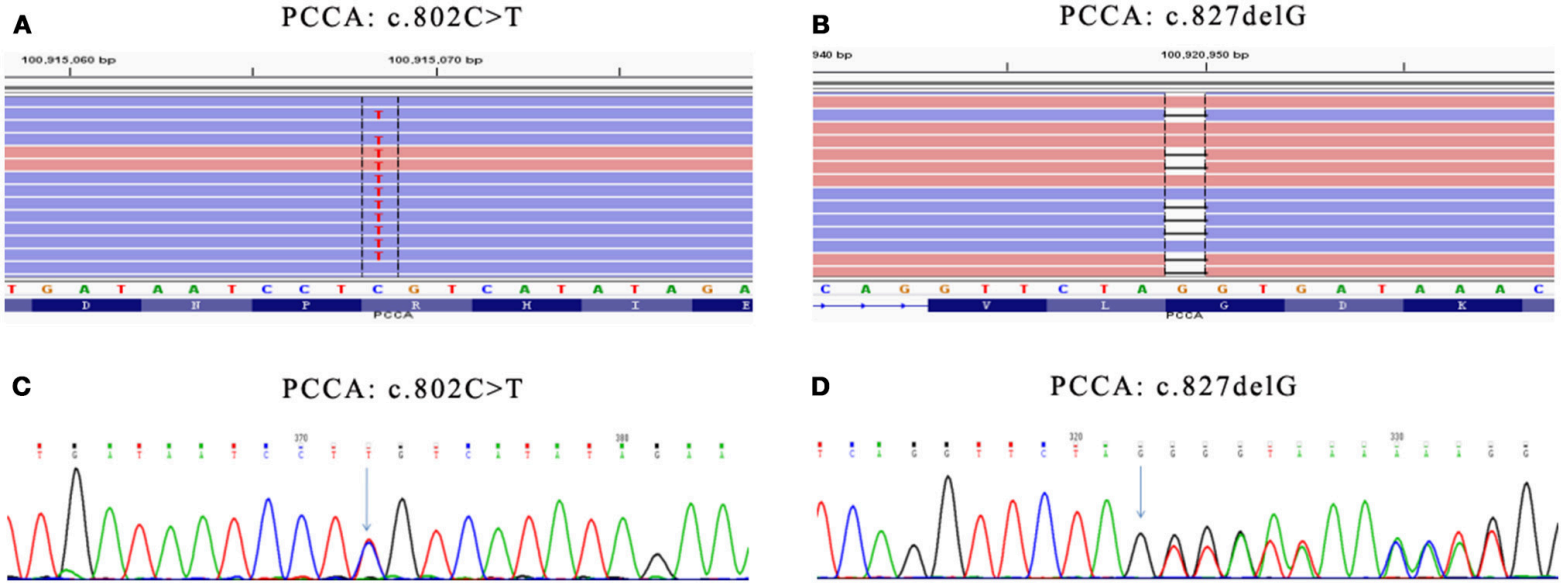
Mutations in PCCA gene. **(A)** Presented in GenBank dbSNP mutation (c.802C>T) in PCCA-exon 10 detected by Genetic diagnosis panel. **(B)** Novel mutation (c.827delG) in PCCA-exon 13 detected by Genetic diagnosis panel. **(C)** Presented mutation (c.802C>T) in PCCA-exon 10 detected by Sanger sequencing. **(D)** Novel mutation (c.827delG) in PCCA-exon 13 detected by Sanger sequencing. The results show father carried c.827delG mutation, while mother carried c.802C>T mutation. Mutation position were marked with green arrow in **(C,D)**.

Genetic diagnosis panel of genetic metabolic disease covers 51 diseases and 98 genes, of which Panel 1 covers 18 amino acid metabolism diseases and 35 genes, Panel 2 covers 17 diseases, and 42 genes of organic acid metabolic diseases and glycogen metabolism diseases, and Panel 3 covers 16 fatty acid metabolism diseases and 21 genes. Genomic DNA was extracted from the peripheral blood of the families using the OMEGA Genomic DNA Extraction Kit (OMEGA Biotech, USA). All mutations were verified by Sanger sequencing. Ion Torrent data extraction, sequence alignment and SNPs and Indels extraction were performed by using Ion Torrent Suite v3.0 software. After the resulting SNPs and indels were filtered by the dbSNP 137 database, HGMD, LOVD and other databases and Pubmed related literatures were retrieved, matching the reported pathogenic sites. Two pathogenic mutations (c.802C>T/c.827delG) were detected in the PCCA gene (Table 2, Figure 1), among them, the variation rs774738181 (c.802C>T) was present on the dbSNP database which appeared to be “Likely pathogenic” in GenBank dbSNP (100915068). we propose that this variation may be pathogenic. (http://www.egl-eurofins.com/emvclass/emvclass.php?approved_symbol=PCCA).

**Table 2:** Genetic diagnosis panel results of genetic metabolic disease Panel.

**Gene**	**Position (hg19)**	**mutation**	**Gene type**	**Amino acid change**	**HGMD ID**	**Mutation type**	**dbSNP**
*PCCA*	chr10:100915068	c.802C>T	Heterozygous	p.R268C	NA	Missense mutation	rs774738181
*PCCA*	chr13:100920949	c.827delG	Heterozygous	p.G276VfsX46	NA	Frameshift mutation	NA

c.827delG was a frameshift mutation, leading to p.Gly276ValfsX46 mutation of amino acid sequence, which has not been reported. According to the ACMG principle, the mutation was a highly reliable pathogenic mutant type. The protein function was predicted by SIFT and PolyPhen, and the results were all harmful. The identified pathogenic mutations and suspected pathogenic mutations were confirmed by Sanger sequencing.

Although two pathogenic mutations were detected in PCCA, two mutations were not reported before, and the 3-Hydroxypropionate in urine was only slightly increased. Therefore, the treatment was postponed and frequency of follow-up was increased.

The patient underwent 1 year of follow-up. MS/MS and GC/MS detection results were shown in Table 3. He remained asymptomatic and his blood ammonia and liver function were normal.

**Table 3:** Detection results of MS/MS and GC/MS in follow-up period.

**Testing time**	**MS/MS**				**GC/MS**
	**C0 (10–50 μmol/L)**	**C3 (0.38–3.6 μmol/L)**	**C3/C2 (0.04−0.2)**	**C3/C0 (0.02−0.17)**	**3-Hydroxypropionate (0–1.1)**
2016.7.13	34.42	3.31	0.34↑	0.1	0.0
2016.8.31	40.06	3.24	0.27↑	0.08	/
2016.9.21	21.52	2.85	0.2	0.13	0.0
2016.11.23	26.48	2.79	0.2	0.11	0.0
2017.5.31	21.98	4.29↑	0.29↑	0.2↑	129.48↑↑↑
2017.6.26	/	/	/	/	15.29↑↑
2017.8.4	29.4	7.03↑↑	0.68↑↑	0.24↑	15.78↑↑

When the child was 1 year old (in May of 2017), C3 and 3-Hydroxypropionate sudden elevated significantly, indicating the pathogenicity of c.802C>T and c.827delG. Thus we suggest that c.802C>T and c.827delG may be linked to late-onset PA.

## Discussion

PCC is located in the mitochondria, and its main biological function is to enable propionyl-CoA carboxylation to form methyl malonyl-CoA. The reduced or lack of PCC activity causes propionyl-CoA accumulation in the body, thereby activating the bypass metabolic pathway and producing a large amount of propionic acid, 3-hydroxypropionate, and methyl citrate. The clinical symptoms of the patients are mainly caused by these intermediate metabolites, and manifested as neurological symptoms such as feeding difficulties, vomiting, lethargy, coma, metabolic acidosis, and hyperammonemia.

Tandem mass spectrometry (MS/MS) enabled Propionic academia (PA) to be early detected based on detecting propionyl carnitine (C3) in neonatal dried blood spot (DBS). American Medical Genetics Society lists PA as one of the 29 diseases to be screened firstly. Neonatal screening laboratory management method issued by China's Ministry of Health in 2017 stipulates that PA is one of the 48 diseases that must be screened. As high C3 was detected in DBS by MS/MS, PA must be differentiated from methylmalonic academia (MMA). The specificity of 3-hydroxypropionate can be detected in the urine of PA patients by GC/MS, while the specificity of methylmalonic acid can be detected in the urine of MMA patients. Therefore, MS/MS and urine GC/MS can make a preliminary clinical diagnosis of patients. C3 in this case was normal in the initial screening and recall check and only manifested as the slight increase of C3/C2, and 3-hydroxypropionate in urine was only slightly elevated. The clinical diagnosis of PA was difficult, thus the pathogenic gene detection was carried out. We used a gene Panel based on NGS technology, and all the exon coverage of PCCA and PCCB gene was 100%.

The PCCA gene is located on chromosome 13q32.3 and contains 24 exons, encoding 703 amino acids. The PCCB gene is located on chromosome 3q22.3 and contains 15 exons, encoding 539 amino acids. Currently, 81 mutations of PCCA and 86 mutations of PCCB have been found, and mutation types have significant differences among different ethnic groups. In Japanese PCCA gene mutations were mainly 923-924insT, IVSl8-6C>G and R399Q, while PCCB gene mutations were mainly R410W, T428I and A153P (3). PCCB mutation in Korean population was mainly T428I, the gene mutation frequency of T428I mutation in PA children was as high as 56.3% (4). PA was common in Greenland Inuit people, and PCCB gene 1540insCCC mutation rate in the normal population was up to 5% (5). People with PA in Latin America had mainly 2 types of mutations in PCCB gene C. 1218-1231dell4insl2 and C. 5029>A, accounting for more than 60% (6).

Two pathogenic mutations (c.802C>T/c.827delG) were detected in PCCA gene of this child patient, c.827delG was a frameshift mutation, leading to p.Gly276ValfsX46 mutation of amino acid sequence, and the mutation was reported by us for the first time. According to the American College of Medical Genetics and Genomics (ACMG) principle, the variation belong to the PVS1 null variant class that can often be assumed to disrupt gene function by leading to a complete absence of the gene product by a lack of transcription or nonsense-mediated decay of an altered transcript. However, the site lacked functional validation. The second variation rs774738181 (c.802C>T) appeared to be “Likely pathogenic” in GenBank dbSNP. Despite the fact that rs774738181 was present on the dbSNP database, we propose that this variation may be pathogenic.

We must be grateful to the cooperation and understanding between the doctor and patient. The child's family members insisted on regular follow-up, even C3/C2 in MS/MS and 3-hydroxypropionate in urinary GC/MS gradually dropped to normal within 6 months after birth. Thus we wondered whether the site is really a pathogenic site. When the child was 1 year of age, C3 and 3-hydroxypropionate concentration began to elevate with the increase in protein intake, and he was diagnosed as PA. This patient should be classified as late-onset case and we suggest that c.802C>T and c.827delG may be linked to late-onset PA.

But the fact which is unable to avoid is, if a deletion is larger than the amplified PCR fragment, or one of the primers for PCR reaction is located at the deleted region, the fragment from the chromosome carrying large deletion will not be amplified at all. Thus, the sequence would appear normal and large deletion would not be detected. For this study the Desviat (7) chose 20 samples of PA patients with no mutations in one or both alleles to study by MLPA analysis. MLPA was employed to screen for deletions in PCCA gene with the SALSA P278 PCCA MLPA kit in these 20 PA patients. Then this strategy revealed a high frequency (21.3%) of deletion alleles, therefore it is very important to perform MLPA analysis for PCCA gene. However, in our department, we also purchased commercial kits directly for MLPA detection, just including SMA, DMD, CAH, PKU, PKD, and Hemophilia. The PA frequency in Nanjing was 2/175160 (Statistical time from December 1, 2013 to July 20, 2018). This patient is the first case of PA, so we had no MLPA kit for PA detention. In follow-up, we will accumulate PA cases and centralize detection by PCCA MLPA kit.

PA lacks specific treatment methods, acute phase is mainly managed by symptomatic treatment. Long-term treatment is to limit the natural protein diet, mainly the intake of valine, isoleucine, threonine and methionine, and is supplemented by levocarnitine, metronidazole, carbamoyl glutamate, and other drug treatment. Recently, liver transplantation is proven as an effective method for the treatment of PA.

This study found that PCCA gene had a new mutation (c.827delG) in a patient with PA, and the clinical follow-up data for the child confirmed that the mutation was pathogenic type. Despite the fact that rs774738181 (c.802C>T) was present on the dbSNP database, we propose that this variation may be pathogenic. In clinical work we must pay attention to the patient's clinical follow-up and doctor-patient relationship. As a rare genetic metabolic disease, PA belongs to the 3-level prevention category of national birth defects. Genetic testing can make clear diagnosis and help diagnosis of pre-symptomatic patient in the family who has not shown onset. In the meantime, genetic testing can be used in prenatal diagnosis. We recommend PA patients should be perform MLPA analysis, and it makes the diagnosis more comprehensive.

## Ethics statement

This study was approved by the local Ethics Committee of Nanjing Maternity and Child Health Care Hospital. Informed written consent was obtained from all patients prior to their enrollment in this study. We sincerely thank all the family members for their participation and cooperation in this study.

## Author contributions

YW and YS conceptualized and designed the study. YW led the review process, drafted the initial manuscript, and YS reviewed all articles and extracted data. TJ analyzed and interpreted the data. All authors made substantial contributions to revising the manuscript. TJ is responsible for the overall content. All authors read and approved the final manuscript.

### Conflict of interest statement

The authors declare that the research was conducted in the absence of any commercial or financial relationships that could be construed as a potential conflict of interest.
